# Effect of hydroxychloroquine blood concentration on the efficacy and ocular toxicity of systemic lupus erythematosus

**DOI:** 10.1038/s41598-024-58391-2

**Published:** 2024-04-01

**Authors:** Peng-Cheng Liu, Shui-Lin Luo, Meng-Na Lv, Yan Wang, Jian-Bin Li, Shu-Jiao Yu, Rui Wu

**Affiliations:** 1 Department of Rheumatology, The First Affiliated Hospital, Jiangxi Medical College, Nanchang University, Nanchang, China; 2 The First Clinical Medical College of Nanchang University, Nanchang, China

**Keywords:** Blood concentration, Efficacy, Hydroxychloroquine, Ocular toxicity, Systemic lupus erythematosus, Rheumatic diseases, Systemic lupus erythematosus

## Abstract

In the absence of evidence-based guidance on the impact of hydroxychloroquine (HCQ) blood concentration on efficacy and ocular toxicity in systemic lupus erythematosus (SLE), the clinical monitoring of HCQ blood concentration is not yet widely performed, which raised concerns about the necessity of conducting HCQ blood concentration monitoring. In this retrospective study, we consecutively enrolled 135 patients with SLE who received HCQ treatment for more than 6 months from July 2022 to December 2022. Ocular toxicity was evaluated by collecting relevant retinal parameters using optical coherence tomography angiography (OCTA). Therapeutic efficacy was evaluated using the SLE disease activity index (SLEDAI) and relevant clinical parameters. HCQ blood concentration was determined by high-performance liquid chromatography-tandem mass spectrometry (HPLC–MS/MS). Spearman correlation analysis revealed that the cumulative dose of HCQ was positively correlated with the foveal avascular zone (FAZ) perimeter and FAZ area (r = 0.734, P < 0.001; r = 0.784, P < 0.001). Meanwhile, the treatment duration of HCQ was positively correlated with FAZ perimeter and FAZ area (r = 0.761, P < 0.001; r = 0.882, P < 0.001). The univariate and multivariate logistic regression analyses indicated that HCQ blood concentration was associated with the disease activity of patients with SLE (odds ratio 0.994, 95% CI 0.990–0.999). HCQ blood concentration may be an important factor in assessing the therapeutic effectiveness of SLE patients. The HCQ-related ocular toxicity was a long-term effect related to long term exposure, rather than the blood concentration of HCQ at the time of testing. More importantly, when addressing HCQ-related ocular toxicity, it may be crucial to pay attention to the cumulative dose and treatment duration of HCQ.

## Introduction

Systemic lupus erythematosus (SLE) is an autoimmune and inflammatory disease. SLE is estimated to have an incidence rate of 47.53 per 100,000 population in China, ranking fourth globally, with a male-to-female ratio of 1:10–12^1,2^. SLE is characterized by the presence of multiple autoantibodies and can affect various systems, ranging from mild skin lesions to severe organ failure. This chronic and recurrent disease can cause irreversible damage to affected organs and may lead to mortality if left untreated or poorly managed.

Hydroxychloroquine (HCQ) is a derivative of chloroquine, an antimalarial drug, in which a hydroxyl group is added to the side chain to decrease its adverse reactions. In the 1950s, HCQ was developed as an immunomodulatory agent and used to treat SLE. Multiple studies have shown that HCQ can reduce disease activity, prevent disease flares, decrease the long-term need for glucocorticoids (GCs), and benefit pregnant women in SLE patients. Furthermore, HCQ has been reported to have multiple comprehensive effects, including anti-thrombotic, hypoglycemic, lipid-lowering, and reduced risk of cardiovascular disease^3,4^. More importantly, HCQ can lower the mortality rate in patients with SLE^5^. Therefore, HCQ is a first-line medication for long-term treatment of SLE, and current guidelines recommend that all SLE patients take HCQ unless contraindicated or experiencing adverse reactions.

Currently, our understanding of the dose exposure/response relationship related to this drug is limited, and there is no consensus on defining the effective blood concentration of HCQ both domestically and internationally. Geraldino's study indicated that HCQ levels fell below 500 ng/mL were associated with higher disease activity^6^, while Durcan believed that an HCQ level above 500 ng/mL was necessary for therapeutic efficacy^7^ and was also a good reflection of patient compliance. The negative predictive value for SLE disease activity was as high as 96% when the HCQ concentration level reached 1000 ng/mL^8^, and Costedoat confirmed that this critical value of 1000 ng/mL could be used as a target concentration for clinical treatment of SLE^9^. However, a multi-center, prospective, randomized controlled trial by French scholars found that an HCQ level above 1000 ng/mL did not reduce the incidence of SLE disease activity^10^.

While the majority of researchers are dedicated to investigating HCQ's therapeutic effectiveness in managing SLE, equal emphasis should be placed on its potential adverse reactions. HCQ's adverse effects encompass a spectrum of issues, including ocular toxicity, skin manifestations, gastrointestinal symptoms, central nervous system manifestations, neuromuscular complications, cardiac-related concerns, and allergic responses. Among these, the most concerning adverse reactions are ocular toxicity, dermatological and subcutaneous manifestations, and cardiac-related complications^11^. The revised recommendations by the American Academy of Ophthalmology (AAO) indicated that the risk of eye toxicities increases with prolonged use of HCQ^12^. However, further research is needed to establish the correlation between HCQ blood concentration and the occurrence of ocular toxicity.

The purpose of this study was to explore the relationship between HCQ blood concentration, therapeutic efficacy, and ocular toxicity in patients with SLE.

## Methods

### Study design and patients

Consecutive patients with SLE were recruited from the First Affiliated Hospital of Nanchang University for participation in this study. All enrolled patients met the following inclusion and exclusion criteria. Inclusion criteria were: (1) aged between 18 and 75 years old, both male and female; (2) met the 2019 EULAR/ACR revised SLE classification criteria^13^; (3) currently using HCQ for at least 6 months; (4) stable daily dose of HCQ for 3 months (100 mg, 200 mg, 300 mg, or 400 mg per day); (5) stable glucocorticoids (GCs) dosage for 3 months; (6) stable dosage of immunosuppressive agents (other than HCQ) for 1 month; (7) willing to sign a written informed consent after being informed of the study. Exclusion criteria were: (1) poor compliance, defined as more than 2 missed doses of HCQ within the past 10 days; (2) presence of retinal lesions (such as macular edema, atrophy, abnormal pigment deposition) or other retinal diseases; (3) allergic to HCQ.

Among the 135 eligible patients, 75 patients underwent Optical coherence tomography angiography (OCTA) examination after being screened according to the following exclusion criteria. Exclusion criteria were: (1) unable to voluntarily and actively cooperate with the examination; (2) had eye diseases such as glaucoma, that could affect the macular area or age-related macular degeneration; (3) had undergone any form of eye surgery in the past; and (4) had concomitant diseases that could affect the fundus or macular degeneration, such as hypertension, diabetes, etc.

### Grouping of patients

The disease activity of SLE was assessed using the SLE Disease Activity Index (SLEDAI) score^14^, evaluated by two different assessors. According to the SLEDAI score, we evaluate the disease activity status of all eligible enrolled SLE patients. The 135 patients who underwent HCQ blood concentration testing were categorized into two groups: the remission group (SLEDAI ≤ 4) and the activity group (SLEDAI > 4). In addition, based on whether patients underwent OCTA examination, the 135 cases were divided into two groups: the OCTA group (n = 75) and the non-OCTA group (n = 60). Finally, based on the different HCQ daily doses, 135 SLE patients were divided into two groups: equal to or less than 200 mg/day group and greater than 200 mg/day group.

### Covariate assessment

Patient demographics (age, sex, height, weight, disease duration), HCQ daily dosage, treatment duration of HCQ, adverse reactions after HCQ administration, concomitant medication (GCs and immunosuppressive agents), laboratory parameters (blood routine, liver and kidney function, blood glucose and lipids, erythrocyte sedimentation rate (ESR), C-reactive protein (CRP), immunoglobulin, complement, and other data were recorded.

### Optical coherence tomography angiography

**Figure 1:**
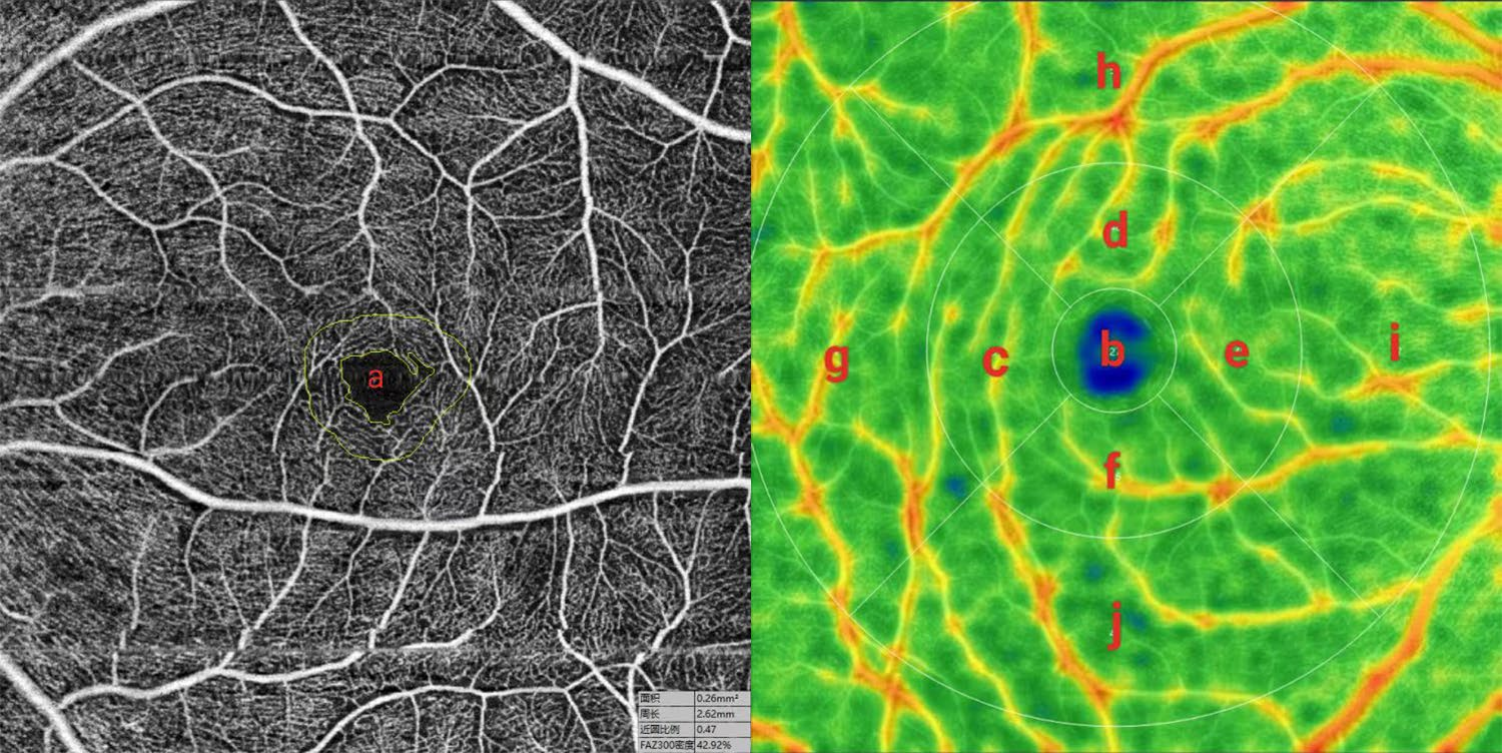
OCTA Parameters in patients with SLE (**a**: foveal avascular zone (FAZ); **b**: macular fovea; **c**: temporal inner macula; **d**: superior inner macula; **e**: nasal inner macula; **f**: inferior inner macula; **g**: temporal outer macula; **h**: superior outer macula; **i**: nasal outer macula; **j**: inferior outer macula).

OCTA imaging of the retinal vessels (SS-OCTA, YG-100 K Yalkaid, TowardPi Medical Technology, Beijing, China) was performed. The patient was advised to use tropicamide eye drops for mydriasis and sit on a chin rest with the head steady. The patient was instructed to gaze at a fixed target within the instrument and avoid eye movement as much as possible. The system automatically captures the images of the patient's macular region, and images with a quality score of ≥ 8 are recorded. If the image quality is not adequate, the scan is repeated. Both eyes were scanned using a 6 × 6 mm macular scan protocol to collect the foveal avascular zone (FAZ) area, FAZ perimeter, superficial capillary plexus (SCP) density and retinal thickness data (Fig. 1). Based on the OCTA results, we assess the ocular toxicity of all patients who underwent OCTA examination.

### Determination of HCQ blood concentration

Two milliliters of peripheral blood were collected from SLE patients using an EDTA (purple) anticoagulation tube and stored at 4 °C before being transported to the Molecular Diagnostic Laboratory of Nanjing Pinsheng Medical Examination Center. The concentration of HCQ in whole blood was measured using high-performance liquid chromatography-tandem mass spectrometry (HPLC–MS/MS).

### Ethical statement

The study was approved by the ethics committee of the First Affiliated Hospital of Nanchang University (IIT2021104) and was conducted by the Declaration of Helsinki (as revised in 2013). Written informed consent was obtained.

### Statistical analysis

SPSS version 26.0 (IBM, Armonk, NY, USA) was used for statistical analysis. Continuous variables were tested for normal distribution using the Shapiro–Wilk test. Normally distributed data were described as the mean ± standard deviation, while nonnormally distributed data as the distributed data or the median (interquartile range). Categorical variables were described as frequencies and percentages. Differences between the two groups were tested by the independent samples t-test or Mann–Whitney *U* test for continuous variables and the chi-square test or Fisher’s exact test for categorical variables. For the comparison of multiple data groups, one-way ANOVA and multiple comparisons were used, and for data that did not obey a normal distribution, the nonparametric Kruskal–Wallis test was used for analysis. Pearson’s test or, where appropriate, Spearman’s test was employed for the correlation analysis. Univariate and multivariate logistic regression analyses were used to assess the relationship between HCQ blood concentration and the disease activity of patients with SLE. All statistical tests were two-tailed, and P < 0.05 was considered statistically significant.

## Results

### Characteristics of all eligible patients

A total of 135 SLE patients were included in the study, including 129 (95.6%) females and 6 (4.4%) males, with a mean age of 40.2 ± 12.1 years. HCQ blood concentrations were assessed in all eligible enrolled patients. The median HCQ blood concentration in the 135 patients was 453.6 (313.8, 621.5) ng/mL. The number of patients receiving daily doses of 100 mg, 200 mg, 300 mg, and 400 mg HCQ were 5 (3.7%), 61 (45.2%), 3 (2.2%), and 66 (48.9%), respectively. The median duration of HCQ treatment was 48 (12, 96) months, and the body mass dependent dose was 4.8 (3.6, 7.5) mg/kg/d, with a cumulative dose of 432 (144, 720) g (Table 1).

**Table 1:** Baseline characteristics of all included patients (N = 135).

Characteristic	Value
Sex	
Female	129 (95.6)
Male	6 (4.4)
Age (years)	40.2 ± 12.1
Duration of SLE (months)	48 (14, 96)^†^
HCQ daily dose	
100 mg	5 (3.7)
200 mg	61 (45.2)
300 mg	3 (2.2)
400 mg	66 (48.9)
Duration of HCQ treatment (months)	48 (12, 96)^†^
Body mass dependent dose (mg/kg/day)	4.8 (3.6, 7.5)^†^
Cumulative dose of HCQ (g)	432 (144, 720)^†^
HCQ blood concentration (ng/mL)	453.6 (313.8, 621.5)^†^
SLEDAI	
Remission	121 (89.6)
Activity	14 (10.4)
GCs	
With	8 (5.9)
Without	127 (94.1)
GCs dosage (mg/day)	6.3 ± 5.0
Immunosuppressant	
With	23 (17.0)
Without	112 (83.0)
BUN (mmol/L)	4.6 ± 1.6
SCr (umol/L)	61.5 ± 17.7
eGFR (mL/min/1.73 m^2^)	106.9 ± 18.5
Immunoglobulin G (g/L)	12.9 ± 3.3
Immunoglobulin A (g/L)	2.6 ± 1.0
Immunoglobulin M (g/L)	1.3 ± 1.1
C3 (g/L)	0.8 ± 0.2
C4 (g/L)	0.2 ± 0.1
ESR (mm/H)	11.0 (6.0, 17.0)^†^
CRP (mg/L)	1.8 ± 2.9

*SLE* systemic lupus erythematosus, *HCQ* hydroxychloroquine, *SLEDAI* SLE disease activity index, *CRP* C-reaction protein, *ESR* erythrocyte sedimentation rate, *C3* complement protein 3, *C4* complement protein 4, *GCs* glucocorticoids, *BUN* blood urea nitrogen, *SCr* serum creatinine.

^†^Nonnormally distributed data were described as the median (interquartile range).

*P < 0.05 was considered statistically significant.

### Comparison of HCQ blood concentration between different groups

**Figure 2:**
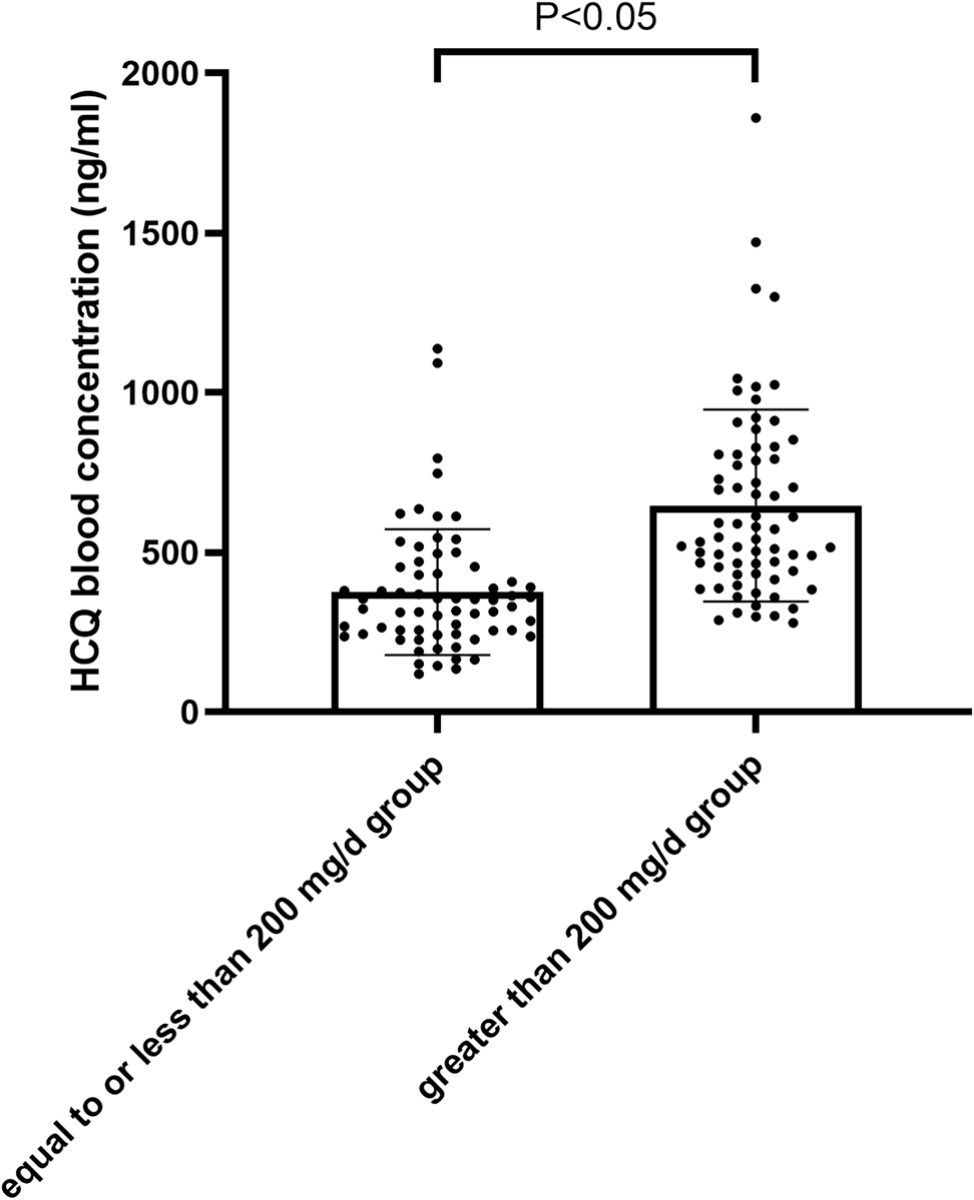
Comparison of HCQ blood concentrations between different daily doses groups.

According to different HCQ daily doses, 135 SLE patients were divided into two groups (equal to or less than 200 mg/day group, greater than 200 mg/day group) and the blood drug concentration of each group was tested. The results demonstrated that the blood concentration of HCQ in the equal to or less than 200 mg/day group is significantly higher than that in the greater than 200 mg/day group (P < 0.05, Fig. 2).

### Correlation analysis of HCQ blood concentration, SLEDAI, and other clinical indicators in patients with SLE

**Figure 3:**
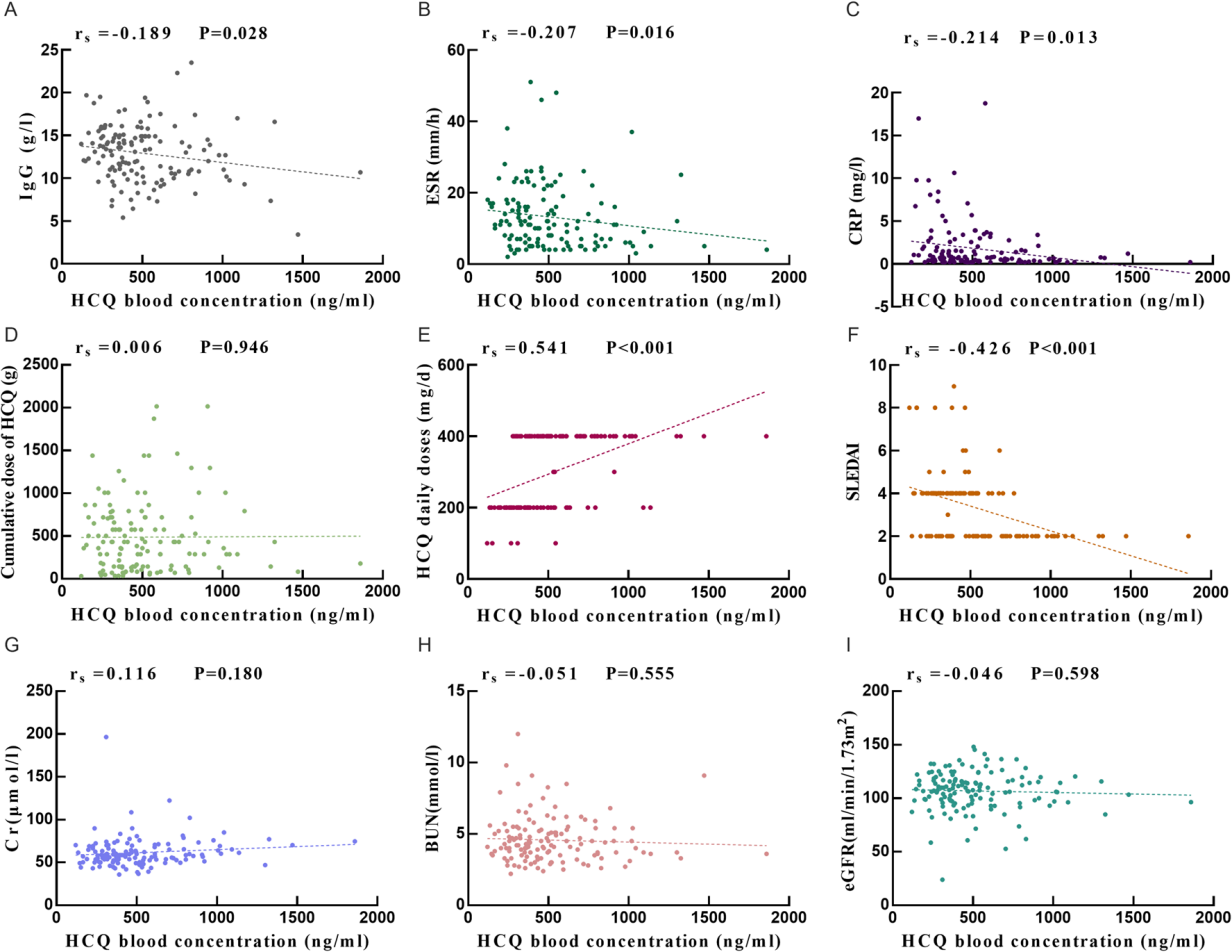
Correlation analysis of HCQ blood concentration with SLEDAI and other clinical indicators in patients with SLE [(**A**) HCQ blood concentration was negatively correlated with IgG. (**B**) HCQ blood concentration was negatively correlated with ESR. © HCQ blood concentration was negatively correlated with CRP. (**D**) There was no correlation between HCQ blood concentration and the cumulative dose of HCQ. (**E**) HCQ blood concentration was positively correlated with HCQ daily doses. (**F**) HCQ blood concentration was negatively correlated with SLEDAI. (**G**–**I**) There was no correlation between HCQ blood concentration and either serum creatinine (SCr), blood urea nitrogen (BUN), or eGF].

Spearman correlation analysis was used to explore the degree of association between HCQ blood concentration and clinical indicators in SLE patients. The results showed that HCQ blood concentration was negatively correlated with IgG (rs = − 0.189, P = 0.028) (Fig. 3A), ESR (rs = − 0.207, P = 0.016) (Fig. 3B), CRP (rs = − 0.214, P = 0.013) (Fig. 3C), and SLEDAI (rs = − 0.426, P < 0.001) (Fig. 3F), and positively correlated with HCQ daily doses (rs = 0.541, P < 0.001) (Fig. 3E). There was no correlation between HCQ blood concentration and the cumulative dose of HCQ (P > 0.05, Fig. 3D). To elucidate the impact of renal function, particularly eGFR, on HCQ blood concentrations, we analyzed the correlation between these parameters. The results found that there was no correlation between HCQ blood concentration and either serum creatinine (SCr), blood urea nitrogen (BUN), or eGFR (P > 0.05, Fig. 3G–I).

### Associations between parameters of HCQ and disease activity of SLE

Using disease activity and remission status as binary outcome variables for patients with SLE, the univariate logistic regression analyses suggested that only HCQ blood concentration might be associated with SLE disease activity (Table 2). In multivariate logistic regression, both Model I and Model II results indicated that HCQ blood concentration was associated with the disease activity of SLE [Model I: OR 0.995, 95 CI 0.990–0.999, P = 0.018; Model II: OR 0.994, 95 CI 0.990–0.999, P = 0.013] (Table 3).

**Table 2:** Univariate logistic regression analysis of clinical biological indicators and the disease activity of SLE.

Parameters	OR (95%CI)	P value
Sex	1.785 (0.193–16.468)	0.609
Age	1.033 (0.985–1.083)	0.177
Duration of SLE	0.996 (0.985–1.007)	0.497
HCQ daily dose	1.001 (0.996–1.007)	0.681
Duration of HCQ treatment	0.994 (0.981–1.006)	0.321
Body mass dependent dose	1.070 (0.832–1.375)	0.599
Cumulative dose of HCQ	1.000 (0.998–1.001)	0.530
HCQ blood concentration	0.997 (0.993–1.000)	0.042*
GCs dosage	0.997 (0.889–1.117)	0.954
Immunosuppressant	0.466 (0.132–1.640)	0.234
CRP	1.110 (0.963–1.278)	0.149
ESR	1.024 (0.970–1.080)	0.394
BUN	1.046 (0.753–1.452)	0.791
SCr	1.008 (0.982–1.034)	0.544
eGFR	0.982 (0.956–1.009)	0.183

*OR* odds ratio, *CI* confidence interval, *HCQ* hydroxychloroquine, *SLE* systemic lupus erythematosus, *GCs* glucocorticoids, *CRP* C-reactive protein, *ESR* erythrocyte, *BUN* blood urea nitrogen, *SCr* serum creatinine.

*P < 0.05 was considered statistically significant.

**Table 3:** Multivariate logistic regression analysis of clinical biological indicators and the disease activity of SLE.

Parameters	Model I			Model II		
	OR	95% CI	P value	OR	95% CI	P value
Age	1.029	0.977–1.085	0.278	0.990	0.916–1.072	0.811
Sex	0.862	0.053–14.090	0.917	0.646	0.037–11.420	0.766
HCQ blood concentration	0.995	0.990–0.999	0.018*	0.994	0.990–0.999	0.013*
Duration of HCQ treatment	0.990	0.962–1.019	0.491	0.981	0.951–1.013	0.237
Cumulative dose of HCQ	1.001	0.997–1.004	0.748	1.000	0.995–1.005	0.972
Daily dose of HCQ	1.003	0.989–1.017	0.675	1.006	0.990–1.022	0.456
Body mass dependent dose	1.149	0.630–2.096	0.651	1.009	0.498–2.045	0.979
Duration of SLE	–	–	–	1.013	0.969–1.059	0.568
GCs dosage	–	–	–	0.995	0.851–1.164	0.951
ISA	–	–	–	0.486	0.112–2.099	0.333
eGFR	–	–	–	0.972	0.930–1.015	0.195

*OR* odds ratio, *CI* confidence interval, *HCQ* hydroxychloroquine, *SLE* systemic lupus erythematosus, *GCs* glucocorticoids, *ISA* immunosuppressant.

Model I adjusted for: Age, Sex, and parameters of HCQ (HCQ blood concentration, Duration of HCQ treatment, Cumulative dose of HCQ, Daily dose of HCQ, Body mass dependent dose of HCQ). Model II adjusted for: Age, Sex, parameters of HCQ (HCQ blood concentration, Duration of HCQ treatment, Cumulative dose of HCQ, Daily dose of HCQ, Body mass dependent dose of HCQ), Duration of SLE, GCs dosage, ISA, and eGFR.

*P < 0.05 was considered statistically significant.

### ROC curve of HCQ blood concentration

**Figure 4:**
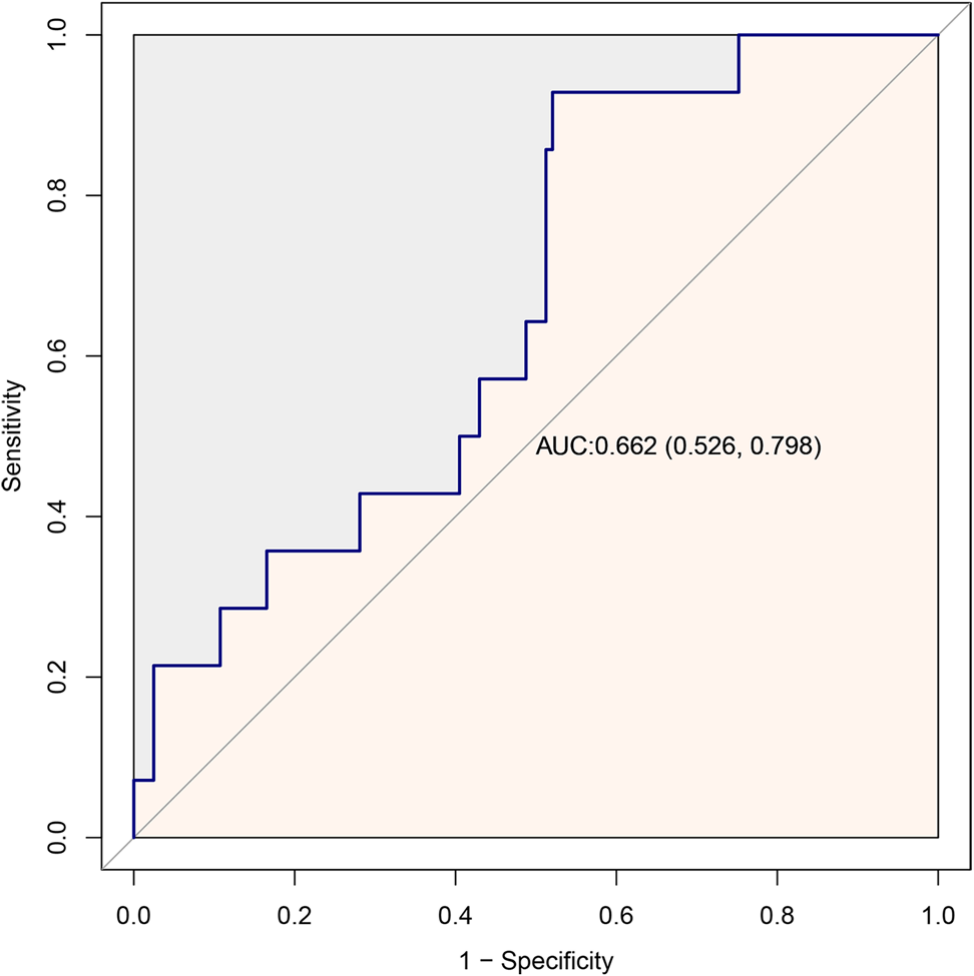
ROC curve of HCQ blood concentration in patients with SLE.

Based on the examination of logistic regression analysis, we additionally established ROC curve of HCQ blood concentration. In this study, the area under the ROC curve was used to indicate the accuracy of HCQ blood concentration to predict the disease activity of SLE. The AUC value for HCQ blood concentration was 0.662 (Fig. 4). Moreover, the cut-off value of HCQ blood concentration was 491.5 ng/mL. The sensitivity and specificity of HCQ blood concentration were 47.9% and 92.9% (Table 4).

**Table 4:** Analysis of ROC curve results.

	AUC	P value	Sensitivity (%)	Specificity (%)	Cut-off value	95% CI
HCQ blood concentration	0.662	0.048	47.9	92.9	491.5	0.526–0.798

*ROC* receiver operating characteristic, *HCQ* hydroxychloroquine, *AUC* area under the curve, *CI* confidence interval.

### Comparison of clinical indicators after grouping based on the cut-off value of HCQ blood concentration

According to the ROC curve results, the effective concentration for SLE remission was determined to be 491.5 ng/mL. To assess the practical clinical utility of the cut-off value of HCQ blood concentration, patients were categorized into a low-concentration group (HCQ blood concentration ≤ 491.5 ng/mL) and a high-concentration group (HCQ blood concentration > 491.5 ng/mL). In the high-concentration group, the SLEDAI score and ESR were lower than those in the low-concentration group (P < 0.05). Additionally, the low-concentration group exhibited lower Hb, PLT, and HDL levels compared to the high-concentration group, with statistically significant differences (P < 0.05). There were no significant differences in the other indicators between the two groups (P > 0.05) (Table 5).

**Table 5:** Comparison of clinical indicators between low-concentration group and high-concentration group.

Characteristic	Low-concentration N = 76	High-concentration N = 59	P value
WBC (× 10^9^/L)	5.75 (3.99, 7.79)^†^	6.23 (5.34, 7.59)^†^	0.088
RBC (× 10^12^/L)	4.26 (3.94, 4.62)^†^	4.37 (4.11, 4.70)^†^	0.193
Hb (g/L)	121 ± 17	130 ± 13	0.001*
PLT (× 10^9^/L)	215 ± 82	242 ± 63	0.034*
ALT (U/L)	15.9 (11.1, 22.2)^†^	15.3 (12.6, 20.4)^†^	0.979
AST (U/L)	20.4 (17.6, 24.0)^†^	19.9 (17.5, 25.4)^†^	0.844
Scr (umol/L)	60.0 ± 12.6	63.5 ± 22.6	0.249
BUN (mmol/L)	4.6 ± 1.5	4.5 ± 1.8	0.707
eGFR (mL/min/1.73 m^2^)	93.3 ± 25.8	91.4 ± 25.2	0.669
Glu (mmol/L)	4.89 (4.55, 5.24)^†^	4.8 (4.57, 5.45)^†^	0.598
TG (mmol/L)	1.3 (1.02, 1.71)^†^	1.27 (0.96, 1.65)^†^	0.765
TC (mmol/L)	4.37 ± 0.94	4.2 ± 0.91	0.289
HDL (mmol/L)	1.18 (1.04, 1.36)^†^	1.47 (1.33, 1.67)^†^	< 0.001*
LDL (mmol/L)	2.42 ± 0.73	2.44 ± 0.76	0.871
CRP (mg/L)	2.2 ± 3.1	1.3 ± 2.6	0.084
ESR (mm/H)	12 (7, 18)^†^	9 (5, 15)^†^	0.033*
Immunoglobulin A (g/L)	2.6 ± 1.0	2.6 ± 1.0	0.867
Immunoglobulin G (g/L)	13.1 ± 3.2	12.7 ± 3.5	0.484
Immunoglobulin M (g/L)	1.3 ± 1.3	1.2 ± 0.9	0.560
C3 (g/L)	0.8 ± 0.2	0.8 ± 0.1	0.728
C4 (g/L)	0.2 ± 0.1	0.2 ± 0.0	0.454
SLEDAI	4 (2, 4)^†^	2 (2, 4)^†^	< 0.001*

*WBC* white blood cells, *RBC* red blood cells, *Hb* hemoglobin, *PLT* platelet, *ALT* alanine transaminase, *AST* aspartate aminotransferase, *Glu* glucose, *TG* triglyceride, *TC* total cholesterol, *HDL* high-density lipoprotein, *LDL* low-density lipoprotein, *SCr* serum creatinine, *BUN* blood urea nitrogen, *CRP* C-reaction protein, *ESR* erythrocyte sedimentation rate, *C3* complement protein 3, *C4* complement protein 4, *SLEDAI* systemic lupus erythematosus disease activity index.

^†^Nonnormally distributed data were described as the median (interquartile range), and the differences between the two groups were tested by the Mann–Whitney *U* test.

*P < 0.05 was considered statistically significant.

### Correlation analysis of OCTA examination parameters, HCQ cumulative dosage, HCQ treatment duration, and HCQ blood concentration

**Figure 5:**
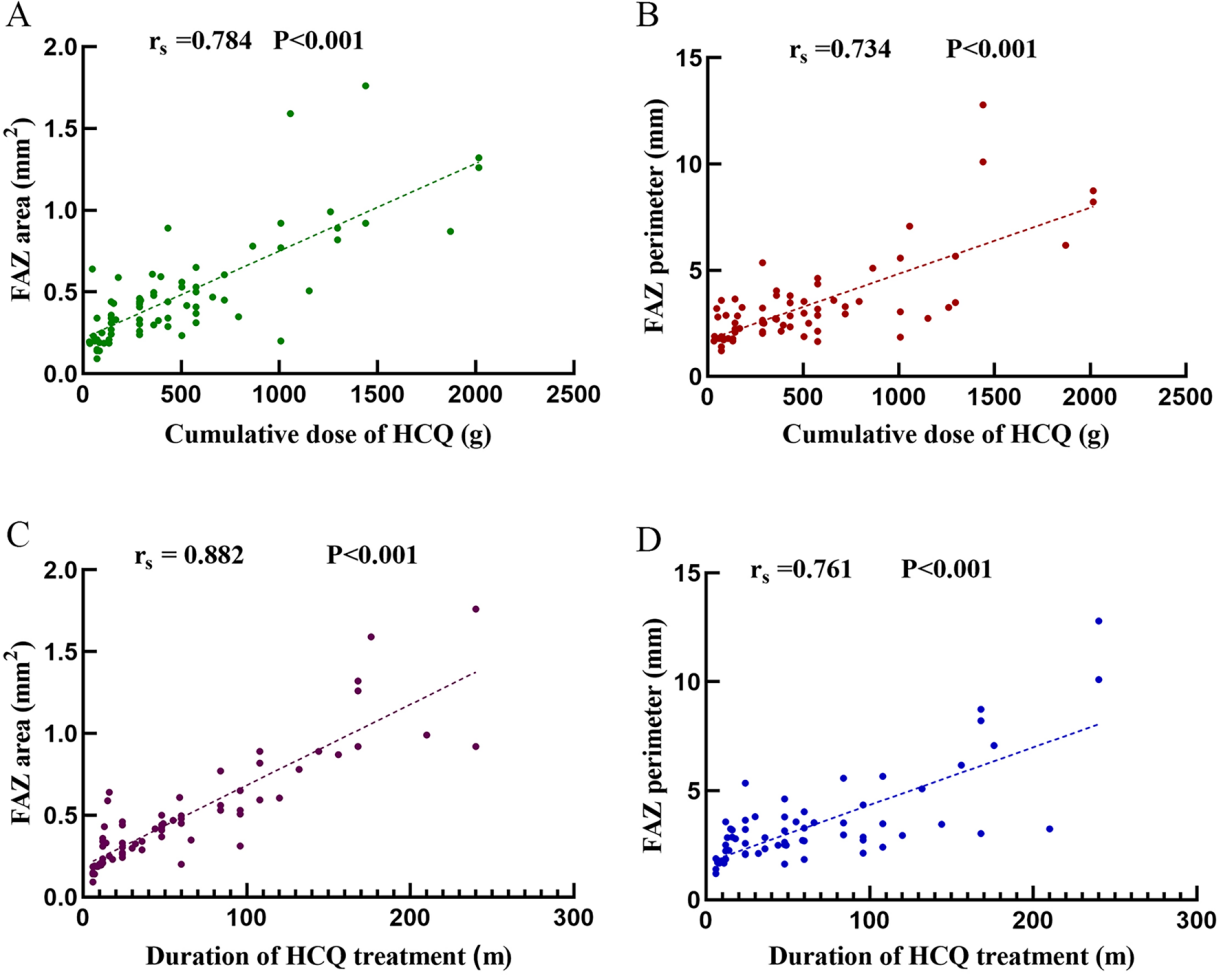
Correlation analysis of OCTA examination parameters with the cumulative dose of HCQ, and the treatment duration of HCQ treatment in patients with systemic lupus erythematosus (SLE). [(**A**) The cumulative dose of HCQ was positively correlated with FAZ area. (**B**) The cumulative dose of HCQ was positively correlated with FAZ perimeter. (**C**) The treatment duration of HCQ was positively correlated with FAZ area. (**B**) The treatment duration of HCQ was positively correlated with FAZ perimeter].

Among 135 eligible patients, 75 patients (150 eyes) underwent OCTA examination. The results showed that the cumulative dose of HCQ was positively correlated with FAZ area (rs = 0.784, P < 0.001) (Fig. 5A) and FAZ perimeter (rs = 0.734, P < 0.001) (Fig. 5B). The treatment duration of HCQ was positively correlated with FAZ area (rs = 0.882, P < 0.001) (Fig. 5C) and FAZ perimeter (rs = 0.761, P < 0.001) (Fig. 5D). Spearman correlation analysis showed no correlation between HCQ blood concentration and either FAZ area, FAZ perimeter, SCP density or retinal thickness (P > 0.05, Table 6).

**Table 6:** Correlation between OCTA examination parameters and HCQ blood concentration.

Parameters	HCQ blood concentration (ng/mL)	
	r_s_	P value
FAZ area (mm^2^)	− 0.033	0.779
FAZ perimeter (mm)	0.012	0.918
SCP density (%)		
Fovea macular	− 0.186	0.110
Temporal inner macula	0.054	0.645
Superior inner macula	0.102	0.383
Nasal inner macula	0.141	0.226
Inferior inner macula	0.047	0.687
Temporal outer macula	− 0.009	0.936
Superior outer macula	− 0.026	0.824
Nasal outer macula	0.092	0.431
Inferior outer macula	0.012	0.917
Retinal thickness (um)		
Fovea retina	− 0.036	0.762
Temporal inner retina	− 0.009	0.941
Superior inner retina	− 0.162	0.164
Nasal inner retina	− 0.094	0.420
Inferior inner retina	− 0.043	0.711
Temporal outer retina	− 0.038	0.742
Superior outer retina	− 0.049	0.679
Nasal outer retina	− 0.055	0.640
Inferior outer retina	− 0.092	0.432

*FAZ* foveal avascular zone, *SCP* superficial capillary plexus.

## Discussion

Our study revealed for the first time that ocular toxicity was not associated with the blood concentration of HCQ. When focusing on the ocular toxicity in patients with HCQ, the cumulative dose of HCQ and the treatment duration of HCQ remain the optimal predictors. Moreover, HCQ blood concentration may be an important factor in assessing the therapeutic effectiveness of SLE patients.

The most common treatment regimen for SLE patients involves a combination of GCs and immunosuppressants. According to the recommendations of the European League Against Rheumatism (EULAR)^15^, the management of SLE should aim to relieve or at least lower disease activity and prevent relapses. HCQ treatment should be given to all SLE patients without contraindications. In the 1920s, chloroquine, a chemically synthesized antimalarial drug, was developed and widely used for the treatment of malaria^16^. However, long-term use of chloroquine can cause various adverse effects, including gastrointestinal symptoms, retinal toxicity, and cardiotoxicity. The appearance of HCQ changed this situation by adding a hydroxyl group to the side chain of chloroquine, which reduces its toxicity. HCQ is currently widely used for a variety of autoimmune diseases^17^. According to the recommendations of the EULAR and AAO^15,18^, the dose of HCQ for the treatment of SLE should not exceed 5.0 mg/kg/day, with a maximum daily dose of 400 mg. In this study, the upper limit of HCQ dose for all patients did not exceed 400 mg/day. The median daily dose based on actual body weight was 4.8 mg/kg/day, which did not exceed the recommended standard.

Previous studies have demonstrated that HCQ can reduce disease activity in SLE. A randomized, double-blind, placebo-controlled study involving 47 patients and lasting for 6 months showed that compared to placebo, taking HCQ reduced the risk of disease flare-ups by 2.5 times in SLE patients^19^. Subsequently, Tsakonas et al.^20^ further expanded on this study and evaluated the risk of disease flare-up after HCQ withdrawal. The results showed that HCQ had a long-term protective effect on major SLE disease flare-ups. A retrospective study by Japanese scholars including 165 SLE patients showed that increasing HCQ treatment can reduce disease activity in SLE patients regardless of background treatment^21^. Our research analysis showed that high HCQ blood concentration level is a protective factor for SLE disease activity, which was consistent with previous studies. Costedoat measured the whole-blood drug level of 143 patients who were taking 400 mg HCQ daily. The mean drug level was 1017 ± 532 ng/mL, and according to the ROC curve, the cut-off value for disease remission was 1000 ng/mL^8^. In our study, involving 135 Chinese SLE patients, the median HCQ concentration in whole blood was 453.6 ng/mL, with a range of 119.9–1859.5 ng/mL, and few patients exceeded 1000 ng/mL. This may be related to differences in ethnicity and dosage. The patients included in Costedoat's study were all Caucasians who received a daily dose of 400 mg. In our study, the daily dosage included 100 mg, 200 mg, 300 mg, in addition to 400 mg. We plotted the ROC curve and the cut-off value for disease remission was 491.5 ng/mL. Interestingly, Durcan et al.^7^ measured HCQ blood concentration in 686 SLE patients and demonstrated a trend for higher disease activity and lower HCQ levels. They believed that an HCQ blood concentration greater than 500 ng/mL could be regarded as the critical value for drug effectiveness, which was consistent with the results of our study.

Ocular toxicity caused by HCQ mainly includes corneal diseases, ciliary body invasion, lens opacity, and retinal lesions, with retinal lesions being the most common. Although the specific pathogenesis remains uncertain, risk factors for HCQ-induced retinopathy include HCQ daily dosage, cumulative dosage of HCQ, treatment duration of HCQ presence of liver and kidney diseases, and the age of patients^22^. Currently, there is no gold standard for diagnosis of HCQ-induced retinopathy. The American Academy of Ophthalmology (AAO) recommends SD-OCT, mfERG, and FAF examinations for screening HCQ users^18^. Overall, the incidence of HCQ-induced retinopathy after long-term use is not high, as evidenced by the retrospective case–control study conducted by Melles et al. on 2361 patients who received continuous HCQ therapy for at least 4 years and underwent SD-OCT examination, which found a total incidence rate of HCQ retinopathy of 7.5%^23^. The relationship between HCQ blood concentration and retinopathy is still disputed. Lenfant's research showed that there was no correlation between HCQ blood concentration and retinopathy, either in single-factor analysis or in multi-factor analysis^24^. However, Petri's prospective cohort study confirmed that the average and peak blood levels of HCQ predicted retinal lesions^25^. The difference in results between the two studies may be due to different definitions of retinopathy. This shows the current research dilemma caused by the lack of consensus on the definition or diagnosis of retinopathy.

In our study, we used an updated examination method-OCTA examination, which is a new non-invasive imaging technology that can generate vascular images in seconds. It has been widely used in retinal vascular diseases and can also be used to evaluate the retinal microvascular structure in SLE patients. Goker et al.^26^ compared the OCTA examination results of 20 patients in the HCQ group with those of 20 patients in the control group, and found that the HCQ group had a significantly larger FAZ area and perimeter than the control group, while the central avascular density of the superficial capillary plexus in the HCQ group was significantly lower than that of the control group. Bulut et al.^27^ confirmed these findings and found a significant positive correlation between the cumulative dose of HCQ and the treatment duration of HCQ and FAZ parameters. Our study results are consistent with Bulut's findings, indicating that the cumulative dose of HCQ and the treatment duration of HCQ are positively correlated with FAZ perimeter and area.

At the same time, we also analyzed the correlation between HCQ blood concentration and OCTA parameters and found no association between drug levels and OCTA examination results. Based on the results of the OCTA examination, we observed that patients who continuously took HCQ for 3 months and achieved a median cumulative dose of 432 (144,720) g did not show significant retinal lesions, indicating that regular dose HCQ has a certain safety profile. More importantly, OCTA parameters were found to be related to the cumulative dose of HCQ and the treatment duration of HCQ, but not to HCQ blood concentration. Therefore, when addressing HCQ-related ocular toxicity, it may be crucial to pay attention to the cumulative dose and treatment duration of HCQ. Furthermore, OCTA can objectively record FAZ and vascular density characteristics before toxicity occurs in patients who are treated with HCQ for a long time and can be used as a screening tool to detect early changes in retinal vasculature.

One of the strengths of our study is the application of OCTA to detect retinal lesions in patients taking HCQ, in addition to the methods recommended by the AAO. Another important strength is that we are the first to combine OCTA parameters with the parameters of HCQ, which not only provides a new method for screening HCQ-induced retinopathy, but also elucidates the correlation between OCTA parameters and the parameters of HCQ. However, our study has some limitations: Firstly, small sample size in patients undergoing OCTA examination might not be powerful to reveal the absolute association between HCQ blood concentration and OCTA parameters. Secondly, the scoring according to our criteria of OCTA may be subjective to some extent, further verification through various detection methods is needed to confirm. Finally, due to the presence of recall bias, there may be some error in the collection of data on patients' HCQ non-adherence. Despite the potential for Type II error resulting from the limited sample size, our findings offer initial insights. However, it is crucial to conduct further studies with larger sample sizes to validate and confirm these results.

## Conclusion

HCQ blood concentration may be an important factor in assessing the therapeutic effectiveness of SLE patients. The HCQ-related ocular toxicity was a long-term effect related to long term exposure, rather than the blood concentration of HCQ at the time of testing. More importantly, when addressing HCQ-related ocular toxicity, it may be crucial to pay attention to the cumulative dose and treatment duration of HCQ.

## Data Availability

The raw data supporting the conclusions of this article will be made available by the corresponding author, without undue reservation.
